# Agri-food data spaces: Highlighting the need for a farm-centered strategy

**DOI:** 10.1016/j.dib.2025.111388

**Published:** 2025-02-18

**Authors:** Gianluca Brunori, Manlio Bacco, Carolina Puerta-Piñero, Maria Teresa Borzacchiello, Eckhard Stormer

**Affiliations:** a Dept. of Agricultural, Food and Agro-Environmental Sciences, University of Pisa, Italy; b European Commission, Joint Research Centre (JRC), Ispra, Italy; c European Commission, Joint Research Centre (JRC), Brussels, Belgium; d Future Impacts, Beethovenstraße 8, Köln, 50674, Germany

**Keywords:** Digital transformation, Data economy, Data governance, Agri-food, Farmers, European Data Spaces

## Abstract

This paper explores the potential of digitalisation in agriculture to improve the sustainability of agriculture production and industrial sectors, contributing to the twin digital and green transition. These systems can facilitate and enhance competitiveness by leveraging on mutually reinforcing transformations. The European Commission has proposed the creation of Common European Data Spaces in specific sectors to support such a transition. We focus on the agri-food domain, considering farmers and other actors in the food chain. The aim is to identify needs, priorities, opportunities, and barriers to a Common European Data Space for agriculture and food systems, thus going beyond the sectoral European Data Space for agriculture already under current development. In addition, this work looks at strategies for introducing the aforementioned novel data space and evidence of benefits for farmers, who are a key component of agricultural and food systems. To accomplish this, the concept of data spaces is presented, analysing main components, functions, and potential challenges and opportunities for data sharing and reuse, with the agri-food context as the main focus. It also presents current and future scenarios for data use at different decision-making levels, focusing on the specific role of farmers in the digital ecosystem. Additionally, it outlines the basic principles for an inclusive agri-food data strategy.

## Highlights

• The European Union (EU) is investing in developing Common European Data Spaces in several domains, including agriculture, pushing for a vibrant data market and data exploitation in the years to come.
• The expected benefits for the farmers, representing key actors in the sector and potential data sources, still need to be further highlighted, calling for more in-depth research.
• To shed some light on such a process, we explore data types, functions, and typologies of users in the agri-food context, having as reference the fast-evolving EU policy framework in the domain of data-related acts.
• We present a use case to connect data and potential users, as well as guiding principles for a data strategy that can benefit different actors in the system.

## 1 Introduction

The future phase of digitalising agriculture and its role in achieving sustainability objectives will depend on the effective utilisation of data. Agricultural data plays a crucial role in ensuring a safe and sustainable supply chain and is essential for transforming food systems [1], [2], [3], [4], [5]. Data collected at the farm level and, more generally, in the agri-food sector, has various uses for both private and public purposes, including farm management, food security, spatial planning, environmental protection, administrative controls, food security, and optimisations in the food chain, among others. The potential providers and users of agricultural data range from farmers themselves to food customers, logistic services, insurance services, controlling bodies, and so on. In the EU alone, there were over 9 million farms in 2020, each engaging in numerous transactions with public administrations, private businesses, consumers, and citizens on a regular basis [6].

The 2021 World Bank’s World Development Report identified three main benefits of data usage: increased accountability, improved policymaking and service delivery, and expanded business opportunities [7]. The effective and efficient use of data used to rely on the establishment of data pools, a concept now challenged by decentralised architectures, in which several actors interested in data exchanges federate together. The challenge in this evolution is not just technological; it also involves developing governance arrangements for data production, storage, integration, and access, following a fit-for-purpose strategy for potential data users. Addressing this challenge requires a social contract between businesses, the government, and civil society, based on three principles: acknowledging the value of data, fostering trust among parties, and ensuring fairness in the distribution of costs and benefits [2], [3], [8].

The EU is leading the way in data policies. The General Data Protection Regulation [9] regulated the use of **personal data**, significantly influencing the operations of businesses and public administrations and setting a *de facto* standard internationally. However, personal data only represent a small portion of the data that comprises the agriculture data economy. Another important category is **open data**, which is generated and held by public administrations and accessible to all. The Open Data Directive [10], which came into effect on 16 July 2019, establishes a framework to promote access and re-use of data held by public administrations, public undertakings, and publicly funded research organisations. This Directive also introduces the concept of high-value datasets (such as geospatial, environmental, and agricultural data), advocating for their availability free of charge in machine-readable formats. A third category is **protected data**, which are not open but can be shared and reused according to specific rules. For example, protected data includes information generated by smart agricultural machinery or those generated through business transactions, such as in the case of agricultural product traceability. In 2020, the European Commission launched the European Strategy for Data [11], with the goal of “creating a single market for data that will ensure Europe’s global competitiveness and data sovereignty”. The strategy aims to address various types of data and data usage, including the use of public sector information by businesses (Government-To-Business –G2B– data sharing), sharing and use of privately held data by other companies (Business-To-Business –B2B– data sharing), use of privately-held data by government authorities (Business-To-Government –B2G– data sharing), and sharing of data between public authorities (Government-To-Government –G2G– data sharing).

In line with the strategy, the Data Governance Act [12] aims to “improve the conditions for data sharing in the internal market, by creating a harmonised framework for data exchanges and laying down certain basic requirements for data governance, paying specific attention to facilitating cooperation between Member States”. The Data Governance Act also defines and regulates the role of data intermediaries, i.e., the providers of data-sharing services. Through the Data Act [13], the EU fosters the development of a framework for data governance, particularly data generated through connected devices (such as precision farming data obtained from Internet of Things devices), and thereby *“contributes to the creation of a cross-sectoral governance framework for data access and use... to provide incentives for horizontal data sharing across sectors*”. A key provision of the Data Act is that “*it imposes the obligation on data holders*^2^ *to make data available to users and third parties of the user’s choice in certain circumstances*”, a rule that potentially challenges data monopolies created by global platforms.

The ultimate objective of this process is to enhance the creation of data spaces where, ideally, data flows freely between individuals and organised users; at the same time, providing clear benefits to interested parties, and especially to the farmers, further than data being easy to access. In its conclusions of 25 March 2021, the European Council recognised “*the need to accelerate the creation of common European data spaces, including ensuring the access to and interoperability of data, and invited the Commission to present the progress made and the remaining measures necessary to establish the sectoral data spaces announced in the European* S*trategy for* D*ata*” [11].

It is worth recalling that data spaces are more than just the technological components, as highlighted in [14], also encompassing technical, organisational, governance, and legal approaches. The interplay of all those together represents the main challenge for data spaces to be adopted and succeed as a strategy.

According to the European Commission’s vision, Common European Data Spaces “*will allow data from across the EU - from the public sector, businesses and individuals as well as research institutions and other types of organizations (e.g. non-profit organisations) - to be made available and exchanged in a trustworthy and secure manner*”.^3^

It is worth pointing out here that data spaces – generically speaking- can be defined as secure and privacy-preserving IT infrastructure to access, process, use, and share data. With Common European Data Spaces, the European Commission refers to data spaces aiming at “*an internal market for data in which data could be used irrespective of its physical storage location in the Union in compliance with applicable law*”. Thus, the emphasis is on the data storage location (i.e., in the EU) and on the internal market (i.e., free flow of data in the EU). Data spaces, and in particular European ones, will need to offer the opportunity to integrate open with protected data in the EU while maintaining the differences in regulation between them. Table 1 provides a list of the most relevant legal acts and policy initiatives on this matter.

**Table 1:** European data-related policy acts (EC stands for European Commission).

Policy Act	Year	Status	Type of act	Type of data	Objectives of the act
INSPIRE Directive	2007, amended in 2019 and 2024	In force	Directive (2007/2/EC)	Spatial data	Establish a European Union (EU) Spatial Data Infrastructure (SDI) to support EU’s environmental policies
GDPR	2016	In force	Regulation (EU 2016/679)	Personal data	Strengthen individuals’ fundamental rights in the digital age and facilitate business by clarifying rules for companies and public bodies
Open Data Directive	2019	In force	Directive EU (2019/1024)	Public data	Stimulate the publishing of dynamic data and the uptake of APIs; limit exceptions allowing public bodies to charge more than marginal costs of dissemination and re-use of their data; enlarge the scope of the Directive to data held by public undertakings and research data resulting from public funding; avoid exclusive private-public arrangements
European Strategy for Data	2020		Communication of the EC (COM/2020/66 final)	Non-personal data	Provides a roadmap to adopt legislative measures on data governance, access and reuse and proposes the creation of a common European data space in 9 strategic sectors
Data Governance Act	2022	In force	Regulation (2022/868)	Protected data in the public sector	Creates the processes and structures to facilitate data sharing by companies, individuals, and the public sector
High-Value Datasets	2022	In force	Implementing Regulation (C/2022/9562 final)	Open data	Public sector bodies holding high-value datasets shall ensure that the datasets are available in machine-readable formats via APIs corresponding to reasonable users’ needs
Data Act	2023	In force	Regulation (2023/2854)	Industrial data	Clarifies who can create value from data and under which conditions
Interoperable Europe Act	2024	In force	Regulation (2024/903)	Open Data	The Act proposes to introduce a structured and co-owned EU cooperation framework for public administrations

The significant effort at the EU level, as evident in Table 1, to regulate data flows has proved valuable. Anyway, “*for smaller companies, the level of complexity due to the interplay of different policies, both at national and at EU level, can be hard to navigate. For businesses that want to participate in data sharing, it should not be necessary to understand all the complexities. Ideally, complying with the data sharing rules should be effortless, even automated*” [15]. This is where Common European Data Spaces can play a role in embedding such complexity and making it effortless for smaller companies, such as most farms in several European regions, to benefit from the data market.

The Common European Agricultural Data Space is one of the data spaces mentioned in the European Strategy for Data [16]. At the same time, several initiatives aimed at building data spaces are in place at different scales, depending on the mix of the actors that compose them and their purposes. For instance, considering agriculture, the Digital Innovation Hub for Agriculture and Food Production Data Space (DADS)^4^ initiative foresees a non-formal network of stakeholders from Slovenia in the field of agri-food. Additional examples are the AgDataHub initiative^5^ in France, the DJustConnect platform^6^ in Flanders, and the Agrifood Data Space Finland (AFDSF)^7^: all represent the willingness to facilitate data sharing in the sector. Benefits can be more or less evident depending on the case, and we can mention here positive examples, such as alleviating administrative burdens (as done by the DJustConnect platform in Flanders); availability of a data-driven climate calculator used to calculate greenhouse gas (GHG) emissions from agricultural products on a farm level (see the case of Agronod^8^); and cross-border data sharing for automated contracts, easier access to machinery data, and improved yields (see the case of the Potato-X initiative^9^). One of the challenges of the Common European Data Spaces will be to take stock of the initiatives already in place and, at the same time, to coordinate the multiplicity of initiatives and ensure their adherence to the objective of providing European citizens with the benefits of the development of data-driven applications while protecting their privacy.

Establishing data spaces involves integrating numerous national, regional, and sector-specific data spaces, creating a complex ecosystem of actors, regulations, infrastructures, and technologies. This process can rely on the initiatives of entities and partnerships of different natures. It involves addressing various obstacles, such as the technological preparedness of involved parties, conflicting interests, lack of trust leading to hesitation in sharing data, technical and organisational challenges, legal barriers, and infrastructure and management costs [17]. Additionally, data spaces can consolidate partnerships between major players - for example, between the input producers and machinery manufacturers- to serve as a significant barrier to entry to other players [18].

Given this context and its intricacies from both a policy and practical perspective, this paper aims to identify the needs, priorities, opportunities, and barriers to establishing data spaces for agriculture and food systems, taking into account the specific characteristics of the agri-food sector, with particular attention to farmers’ needs who are often the weakest link in the chain. We will proceed as follows: Section 2 illustrates the methodology followed; Section 3 identifies the main concepts related to data spaces, in general; Section 4 discusses the role of farmers in the agri-food data space; Section 5 identifies the principles for a strategy of developing an agri-food data space. Concluding remarks are in Section 6.

## 2 Methodology

To develop the proposed framework, the authors have collected and analysed, between 2021 and 2024, information from various sources. Through explorative bibliographic search, the authors created a map of relevant concepts related to the understanding of ’agri-food data spaces’, which form the basis of this paper’s narrative. Subsequently, they conducted keyword searches based on this map, focusing on the most frequently cited papers in each section. Additionally, the authors reached out to approximately 52 experts and stakeholders, engaging in in-depth interviews, focus groups, workshops, personal discussions, and field visits, some of which were conducted as part of projects to which the authors participated.^10^ The results of the Coordination and Support Action (CSA) AgriDataSpace^11^ have been taken into consideration. These studies focus on the contributions and limitations of digitalisation for reducing GHG emissions and increasing resilience in agriculture; data spaces are identified as an enabling intermediary.

## 3 Data spaces: concept and conditions for development

Before delving into the case of agri-food, we provide a brief introduction to the concept of data space from a technical standpoint. According to The Big Data Value Association (BDVA), data spaces encompass an umbrella term corresponding to any ecosystem of data models, datasets, ontologies, data sharing contracts, and specialised management services (i.e., as often provided by data centres, stores, repositories, individually, or within a ’data lake’), together with soft competencies around it (i.e., governance, social interactions, business processes) [19].

Technically speaking, a data space represents the software infrastructure to interconnect data sources. By federating data sources using specific software components, namely connectors, their content can be shared, exchanged, and accessed in an interoperable manner, paving the way for the development and use of data-driven applications and services. Holders and producers of data can establish rules, such as limiting access to authenticated and authorized parties. Access to data can be subject to the payment of fees. To summarise, the central concept of data spaces is *connection*, as tools and rules are developed to interconnect data of different natures. This concept moves beyond the top-down standardisation that characterises initial attempts to pool data, for example in the INSPIRE initiative and all traditional Spatial Data Infrastructures [17], towards more flexible approaches. The potential societal benefits of data spaces are linked to the accessibility and reusability of data. In a data space, ecosystem components work to ensure that data adhere to FAIR principles, i.e., Findable, Accessible, Interoperable, and Reusable [20]. Among these properties, interoperability presents the greatest challenge -especially for the agricultural sector-, encompassing technical, semantic, organisational, and legal dimensions [21].

Collecting and organising data through data spaces requires consideration of different use cases. The same data can serve multiple purposes; for example, soil data can aid farmers in predicting yields, selecting which crops or varieties to grow, and determining fertiliser usage. The same data, when aggregated with data from other farms, can be of interest to agricultural trading companies for estimating potential supply, or for public authorities to monitor soil health. However, contextualisation of data and metadata are crucial elements as well.

**Fig. 1:**
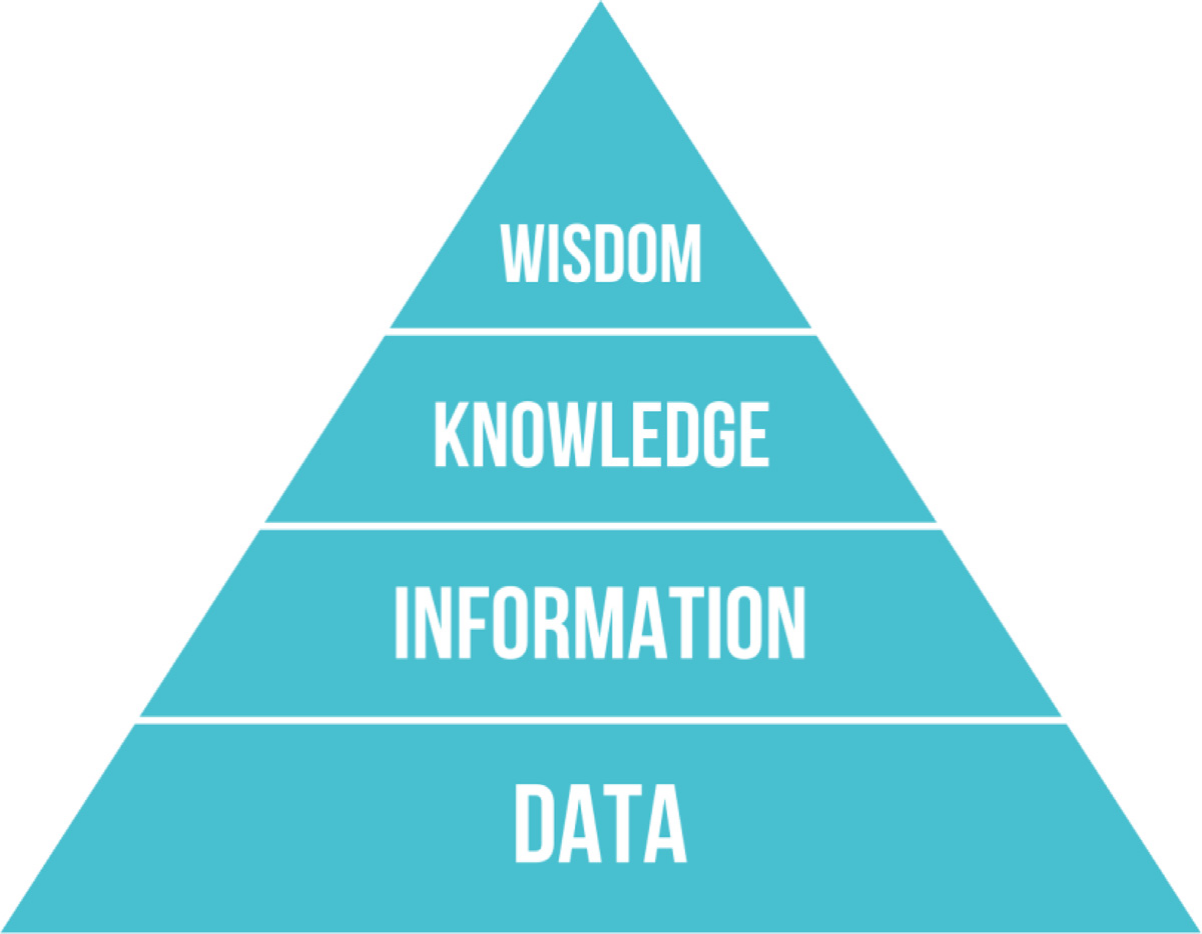
Ackoff’s knowledge hierarchy (adapted from [22]).

To discuss the role of data in the creation of value, we use the concept of knowledge hierarchy (Fig. 1). Proposed in [22], the model helps to provide insight into the manifold role of data spaces for agriculture and rural development. In Ackoff’s model, data are products of observation and sensing; they represent the properties of objects. However, the accuracy of this representation depends on the size of the sample, the frequency, the sensitiveness of the instruments, and the components of the environment that are left out of the field of observation. To become information, data should be linked to a context, by adding metadata. Translating data into information requires effort. In precision farming, given that sensors capture only part of the environmental components, data are used to e.g., feed models that require calibration. Only after calibration, data can provide information.

Information is an input to decisions. However, to be useful, information must be evaluated. A cloudy sky can provide information to the farmer about imminent rain, but whether this information is valuable or not will depend on the farmer’s knowledge. Therefore, knowledge consists of rules that help to evaluate information. Finally, wisdom is the capacity to adapt data, information and knowledge to the specific context of action, and to make decisions. The transformation of knowledge into wisdom, therefore, does not depend only on information but is based on experience and interpretation.

While connecting and interoperating the different levels of the pyramid for different purposes and users, new emerging needs, challenges and opportunities may also arise. In the end, data spaces can be thought of as environments designed to make the transition from data to knowledge (and vice versa) easier for all users in a multiplicity of domains.

Furthermore, agri-food data spaces must integrate both open and protected data to create opportunities for novel services to public administrations, private businesses, and civil society while also safeguarding the privacy and legitimate interests of individuals and businesses and preventing the concentration of power. The challenges experienced in the implementation of the INSPIRE Directive [23] demonstrate that the integration process is complex and requires substantial human effort, relevant skills, financial costs, and political negotiation, among other factors. In fact, a key lesson learned during the INSPIRE implementation is that the process should be demand-driven, fuelled by the evidence of benefits of having access to data and by the initiatives of actors who aim to gain this value [24]. In this regard, a better understanding of the utility that the data can generate in the agri-food system is of utmost importance.

## 4 Building Agri-food Data Spaces: Analysing the Components

### 4.1 Types of agri-food data

An agri-food data space could contain data with different content and generated from a variety of sources. Part of these data are collected for general purposes, as in the case of Earth Observation (EO) and weather data. For example, with EO it is possible to get information on land use, availability of water, soil quality. Other data are generated when transactions occur - marketing communication, buying and selling, administrative procedures, and farm operations - between actors of the relevant ecosystem. In Table 2 we distinguish: a) data related to farm structures; b) process-related data; c) administration data; and d) communication data.

**Table 2:** Classification of transaction-generated data.

	Within farm	With government	With other business	With consumers
Farm structures	Personal data; farm layout; equipment	Land tenure; buildings; machinery; livestock	Data collected by consultancies	Signalling environmental and landscape anomalies
Farm processes	Farm environment; process; product; labour effort; inputs; costs; incomes	Inspection; veterinary services	Precision farming apps; selling and buying; certifications; quality checks; traceability	Signalling illicit; frauds
Farm administration	Internal transactions	Application for subsidies; payments; certifications	Orders; payments; invoices	Online search product usage
Farm communication	Internal communication	Administrative communication	Advisory services; peer to peer	Marketing; public relations; user experience

Digitalisation has the potential to significantly increase the volume of available farm data. Software applications can store and process data, simplifying retrieval, analysis, and sharing. However, transitioning from paper-based to digital data management may present a barrier for some farmers who may lack the necessary skills, time, means, or motivation. Sensors have the potential to automate data collection, but not all sensor-generated data can be immediately transformed into usable information. The Internet of Things (IoT) concept facilitates the connectivity of various devices, including those on board smart machinery, leading to advancements in the integration of diverse data sources. In agriculture, sensor-generated data can be categorised as in situ and remotely collected. In situ data is gathered either by direct observation in the field (as the state of the phenology of the crop or the presence of plagues or pollinators) or by using smart machinery and environmental sensors, providing insights into e.g., weather, soil conditions, plant and animal welfare, production processes. Remote data collection uses sensors installed on remote infrastructures such as satellites, manned, and unmanned aerial vehicles, which can provide information on farm structures and environmental conditions. Further, farmers and other actors, such as food operators and authorities, collect and create relevant data for business, policy, and consumer purposes. For example, farmers can hire machinery services that collect data when they carry out farm operations; veterinaries collect livestock-related data with their instruments for diagnostic purposes; farm advisors monitor the development of crops to provide advice; farm associations keep administrative data on behalf of farmers. Food operators must keep data related to critical points in food-related processes for hygiene, safety, and environmental reasons might also generate more often in situ data.

Data can be analysed by farmers and their consultants to generate spatial maps displaying agro-ecological characteristics such as soil fertility, crop productivity, and water requirements; association of input data with crop performance data provides insights on input performance; aggregated data can be used for Research and Development purposes to develop new models and new Artificial Intelligence (AI) tools; public administrations could use these data for administrative control or policy evaluation (e.g., the different paying schemas of the Common Agriculture Policy, CAP).

Farm data are managed at various spatial and granularity levels, including the farm and the supply chain, from the farm to regional, national, and EU levels. For instance, national or regional databases certify land ownership and record the history of land tenure, while surveys and censuses contribute statistical data for policymaking. Integrated Administration and Control System (IACS) stores administrative data related to farms, forming the basis for CAP information systems. Additionally, EO systems using satellite remote sensing platforms provide data at a planetary level, paving the way for specialised platform providers and data service providers.

While the availability of open data is somehow important, ensuring its findability and interoperability is equally important. For example, it is increasingly crucial to interconnect and easily discover data on logistic operations of agricultural products along the food chain with spatial food availability and spatial location of the food/input industry. Moreover, the success of data spaces depends on easing data collection and management for all participating actors without relinquishing control over data.

### 4.2 Functions of agri-food data

Data serves different functions and holds varying values depending on the users. Business users utilise data to enhance process efficiency, develop new products and services, and implement marketing strategies. Civil society users rely on data to increase awareness of publicly relevant facts and contribute to public discourse [25]. Public administrations require data for policy-related decision-making. However, in some instances, granting data access to one entity (e.g., public administrations) may pose risks to other parties (e.g., potential sanctions following audits). Research organisations can benefit all user categories depending on the research objectives, and sharing research data can facilitate rapid responses to emerging issues [26].

Crucially, different purposes necessitate data in distinct formats or pre-processing states. For example, regulatory bodies require different information than the food industry, while farm-level task automation and decision-making support tools demand highly specific data to operate effectively [27]. This creates opportunities for various data intermediaries with specialised roles such as data aggregation, cleaning, integration, and processing [28].

The potential unequal distribution of costs and benefits of data availability underscores the need for negotiated and regulated data sharing and access. For all four user categories, data functions can be categorised into three groups: monitoring, decision support, and communication (see Table 3).

**Table 3:** Functions of data and typologies of users.

	Research		
	Business	Civil Society	Policymaking
Monitoring	Business activities; business environment; operations	Business and public administrations’ societal impact; state of the constituencies	Effectiveness and efficiency of policies; state of agri-food system
Decision Support	Operations; strategy; product development	Civil society’s initiatives	Policy design; policy implementation
Communication	Cooperation; marketing; public relations; accountability	Advocacy; lobbying; protest; participation; consensus building	Governance, accountability; policy enforcement

Monitoring is the gathering of information on a specific object or activity and the assessment of critical variables over time. Monitoring is key to more efficient processes, as it provides feedback on actors’ choices and allows assigning responsibilities. Decision support refers to the prediction of critical events in a system, so to help decision-making. Decision support is based on the construction of representations of the observed objects and on theories, models and heuristics that help to link the state of a variable to its potential outcomes. Communication refers to the possibility of activating transactions and sharing information between actors of a system. Digital communication also allows the creation of new organisational forms, new services, and new business models.

In the following sections, we explain the relevance of data functions for each category of users, focusing on the agri-food sector.

#### 4.2.1 Data for business

Within the business category, we consider farms, food processing and trading firms, as well as suppliers of supporting materials such as agrochemicals, feed, and machinery [29]. The digital transformation in the agri-food sector has brought visible advantages in the domain of communication, affecting logistics, commercial transactions, administrative and financial operations, organization, and decision-making [30]. Digital communication has revolutionised supply chains through disintermediation, allowing farms to have visibility on the market and interact with consumers [31]. Machinery manufacturers and input providers are transforming their business models as they can sell data-related services that support farmers in the optimisation of their operations. The number of wireless devices installed worldwide in agriculture, estimated at around 25 million in 2022, is forecasted to grow to 37 million in 2027 [32]. The global market size of smart agriculture is expected to grow from approximately 15 billion U.S. dollars in 2022 to 33 billion U.S. dollars by 2027 [33].

The process of datafication of agri-food processes is closely linked to the function of monitoring [34]. Farmers can monitor various aspects such as the state of the soil, animals, plants, product levels and quality, as well as the movements of animals and machines [35]. Data availability can generate irrigation services to fertilising or pesticide/insecticide solutions. Landowners could be interested in productivity data. Financial institutions need data access to assess farm businesses [36]. Processors would benefit from a timely update on the production potential of their suppliers and the quality characteristics. However, challenges exist in terms of accuracy [37].

The quantity of data provided by sensing technologies is not manageable without digital tools, and decision-making relies on the capability to turn data into easy-to-read information [38]. The economy of data grows when monitoring, decision support, and communication are interlinked, with the ability to access external databases and share data from various sources to support decision-making [39]. Platforms and gateways to global but specialised networks are growing, generating a process of re-intermediation and building ecosystems of actors with complementary needs [40], [41]. The rise of agricultural platforms that transform data into decision-support knowledge is evident, and open data sharing is also growing to enable digital business transformation [42], [43].

Along with opportunities, the digitalisation of the business sector presents several emerging threats, including those based on design, access, and complexity [29], [44]. These threats encompass issues such as bias generated by AI, digital divides and concentrations of power, and systemic unexpected consequences and loss of control in processes by human actors.

#### 4.2.2 Data for civil society

Data spaces will play a crucial role in sustainable food systems by reconfiguring the relationships of civil society, businesses, and the State, providing essential information to understand the production and impact of food. Food safety and security as well as food quality will be highly impacted using data [45]. Data availability can help civil society groups monitor the impact of farms on the environment and their production of public goods. Supporting to measure the product footprint, data make responsible consumption and production patterns possible. Data can also provide information on the potential trade-offs or conflicts with environmental or climatic goals of the sustainability of the food systems along the supply chain [46]. The availability of agri-food data at the business unit level allows easier and more accurate environmental footprint indicators and sustainability business reports. The availability of data collected for business purposes might change the methodologies of collection and release of statistical data. However, concerns about misinformation, disinformation, and control of data remain [45], [47].

#### 4.2.3 Data for policymaking

The increased accuracy and frequency of data collection through digital systems have extensive applications in planning, risk management, policy design, and enforcement [48]. These digital technologies also allow for the integration of agricultural data with environmental conditions and infrastructure needs, reducing the administrative burden on public authorities and providing tools for policy monitoring and assessment [49], [50].

Monitoring systems can provide timely information on weather conditions, prices, crop adversities, and even farmers’ stress, enhancing the accuracy of prediction models and supporting the transformation of sustainable systems in view of the UN Sustainable Development Goals [2], [3], [8], [51]. Additionally, improved farm-level data and EO data pave the way for policies over large spatial scales, addressing the need for landscape-level agri-environmental policies based on collective action [52]. The development of data spaces can also foster new methodologies for collecting statistical data.

However, the use of data for policy purposes can generate challenges and unintended consequences. Increased control capacity from public administrations can lead to a risk-averse approach by farmers in sharing data, raising concerns about privacy and reputation [53]. Yet, a collaborative approach aimed at sharing monitoring data with farmers can lead to mutual learning and improved communication between stakeholders [48].

Digital technologies can also assist in policy evaluation by gathering new knowledge and allowing for improved analysis, potentially facilitating a wider range of policy impacts and improving the robustness of policy evaluations [31]. However, especially looking at policymaking, one of the key issues in using data is data quality, as new data sources may be limited by selectivity, coverage bias, and unreliable methodologies [54], [55] and thus affect payments, sanctions, and information to beneficiaries.

#### 4.2.4 Data for research

Digitalisation is revolutionising research practices and fostering enhanced collaboration within and across international networks [56]. Research organisations are sharing resources, infrastructures, and findings, leading to an increase in co-authored research products and the circulation of knowledge at negligible costs. Access to bibliographic databases allows for meta-knowledge development, facilitating interdisciplinary work and the easier exchange of research questions and results through specialised social media [57]. This transformation in research practices is contributing to the transition to Open Science, which promotes the unrestricted circulation and sharing of knowledge, challenging the traditional paradigm of science and innovation built on privileged access to knowledge [58]. Open Science is increasingly supported by public research policies and is also gaining traction in private research and public-private collaborations, as it is recognised that collaboration and sharing are essential for accelerating innovation. Open research and innovation create knowledge ecosystems where cooperation and competition coexist, fostering the emergence of new insights and discoveries.

Data plays a crucial role in Open Innovation, serving as a strategic resource for effective and timely research results [59]. The access to new sources of data, including those collected by citizens, has paved the way for new approaches such as Citizen Science. However, the transition to Open Science necessitates specific policies to regulate data management and facilitate the sharing and reuse of research data [60]. Initiatives like the European Open Science Cloud (EOSC)^12^ are at the forefront of these efforts, aiming to develop a ’Web of FAIR Data and services’ for science, research, and innovation data space, ranging from visualisation and analytics to long-term information preservation.

The development of European Data Spaces can make an important contribution to Open Innovation. Research can be one of the main drivers of the development of data spaces, as it develops data collection methodologies, identifies new relations between signals and events, develops analytical tools, provides new hardware and software for data management, and improves the methodologies to make existing datasets open and interoperable. Moreover, research is a potentially endless source of elaborated data and proof of concepts that could enhance also the data value along the food and decision-making chains. Although the goals of Open Innovation and Data Spaces are not necessarily the same, we believe that the potential of data sharing can create a convergence in the long run.

## 5 The Role of Farmers in Agri-food Data Spaces

Many actors within the food system are aware of the advantages that an agri-food data space may provide, such as optimising data management, improving value chain strategies, and providing adequate information to consumers and the public. Data spaces can generate new data-related services, nurturing new digital ecosystems. Public authorities could also benefit from more effective and less costly controls, while civil society could have access to information on the contribution of the food system to the common good. For research, access to data spaces would open a multiplicity of new avenues.

On the contrary, farmers’ awareness of the added value of these data is still low, partly because the goals of data collection are typically set by more powerful actors, and partly because farmers’ involvement during the initial phases of developing data-related tools is limited. The risk of creating data spaces without an active involvement of farmers is that data-related solutions are poorly equipped to address farmers’ needs, or that data-related solutions are tailored only to specific categories of farmers.

Existing attention on data spaces in agriculture has been so far mainly centred on precision farming, pushed by key players such as machinery producers and input providers. Although precision farming will have a growing importance in the future, we cannot forget that farmers already collect a substantial amount of data without “smart tools”, for example as a requirement for receiving CAP subsidies, to comply with health authorities’ requests for information about critical points of farming processes, to adhere to quality schemes. These data could be used not only to improve productivity and efficiency at the production level but also to create value along the supply chain. However, collecting and managing this data is often burdensome for farmers, who enjoy a limited return on their efforts.

The creation of value from data will depend also on the access farmers will have to data and their power relation with data holders.^13^ As the Data Act points out, “*in sectors characterised by the concentration of a small number of manufacturers supplying connected products to end users, there may only be limited options available to users for the access to and the use and sharing of data. In such circumstances, contracts may be insufficient to achieve the objective of user empowerment, making it difficult for users to obtain value from the data generated by the connected product they purchase, rent or lease*.” Further than that, it states that “*this Regulation should therefore build on recent developments in specific sectors, such as the Code of Conduct on agricultural data sharing by contract. Data holders should not use any readily available data that is non-personal data to derive insights about the economic situation of the user or its assets or production methods or about such use by the user in any other manner that could undermine the commercial position of that user on the markets in which it is active*.” And, finally, that “*the user should be given the necessary technical interface to manage permissions, preferably with granular permission options such as ‘allow once’ or ‘allow while using this app or service’, including the option to withdraw such permissions*”.

To encourage farmers to share and use their data, agri-food data spaces should be designed with the purpose of maximising benefits for farmers, addressing their concerns, and providing incentives for data sharing. Digital technologies have the potential to alleviate the burden of collecting and keeping records, thereby making data collection easier and more efficient for farmers. This could imply working to make data collection easier, striving for increased interoperability of databases, and making it possible for farmers to sell their data. Additionally, it would provide access to retailers’, processors’, and public administrations’ data and could support farmers’ decision-making, if there are clear rules that forbid using data to harm farmers directly or indirectly.

### 5.1 Farmers’ role in the data space: a use case

Antonio operates a sheep farm in the hilly regions of southern Tuscany, encompassing 50 hectares of land and a herd of 150 predominantly grass-fed sheep. In favourable weather, the sheep graze in the pastures daily, but during winter when the pasture grass is insufficient, Antonio supplements their diet with farm-grown hay and purchased cereals due to persistent drought causing summer pasture shortages.

Newborn lambs must be registered within one week of birth in the livestock registry, with earmarking for identification, but Antonio often delegates this task to the producers’ association technicians. Consequently, due to unattended lambing, he sometimes struggles to identify genetic lines. To optimise pasture resources, Antonio partitions the land with mobile fences and rotates the sheep across different sectors to prevent overgrazing, relying on experience rather than data on area productivity.

Employing a low-input method for crop cultivation, Antonio receives EU CAP subsidies, thus he must maintain a registry of fertiliser and pesticide applications, backed by input purchase invoices, and share this data with administrative authorities through the local farmers’ organisation.

As the sheep have free movement between the pasture and barn, Antonio manually records daily sheep counts during milking and veterinary treatments in separate registries. Milking is automated, with collected milk stored in a refrigerated tank and collected daily by the cooperative. The cooperative maintains records of Antonio’s daily milk production and composition, which forms the basis for payment, shared monthly via paper letter. Additionally, the cooperative processes Antonio’s milk into a Protected Designation of Origin (PDO) cheese, requiring Antonio to maintain a registry of purchased feed to ensure compliance with PDO rules.

The verification of this feed information by a certification body allows the cooperative to label the cheese as PDO, commanding a premium price due to its assurance that it is produced with sheep milk from grass-fed sheep in Tuscany. Table 4 outlines the datasets and collection methods relevant to the use case described above [61].

**Table 4:** Data collected at farm level in a sheep farm.

ID	Dataset	Data	Method of data collection	Information	Compulsory
1	Stable registry	Identifier, date of birth, death, purchases, sales	Manual	Daily size and composition of the herd	Yes
2	Georeferenced soil data	Date, geographical coordinates light absorbance	Satellite, drone (using sensors)	Soil maps	No
3	Genealogical book	Parents of newborns	Manual	Genealogical series	No
4	Milk production	Sheep id, date, milk produced	Manual, digital	Times series of production	Yes (aggregated)
5	Milk quality	Date, quality of milk	Milk chemical analysis	Daily quality, time series	No
6	Registry of treatments	Date, pharmac., treated animal	Manual	Time series	Yes
7	Registry of fertilization and pest management	Date, fertilisers, pesticides, parcels	Manual / precision sprayer	Time series	Yes
8	Registry of feed	Date of purchase, seller, type of feed	Manual / barcode scanner	Quantity and quality	Yes
9	Registry of sales	Farm id, date and quantity of milk sent to cooperative	Manual / digital balance	Time series	Yes
10	PDO certificate	Farm id, date, declaration of compliance	Manual	Compliance with PDO	Yes

**Fig. 2:**
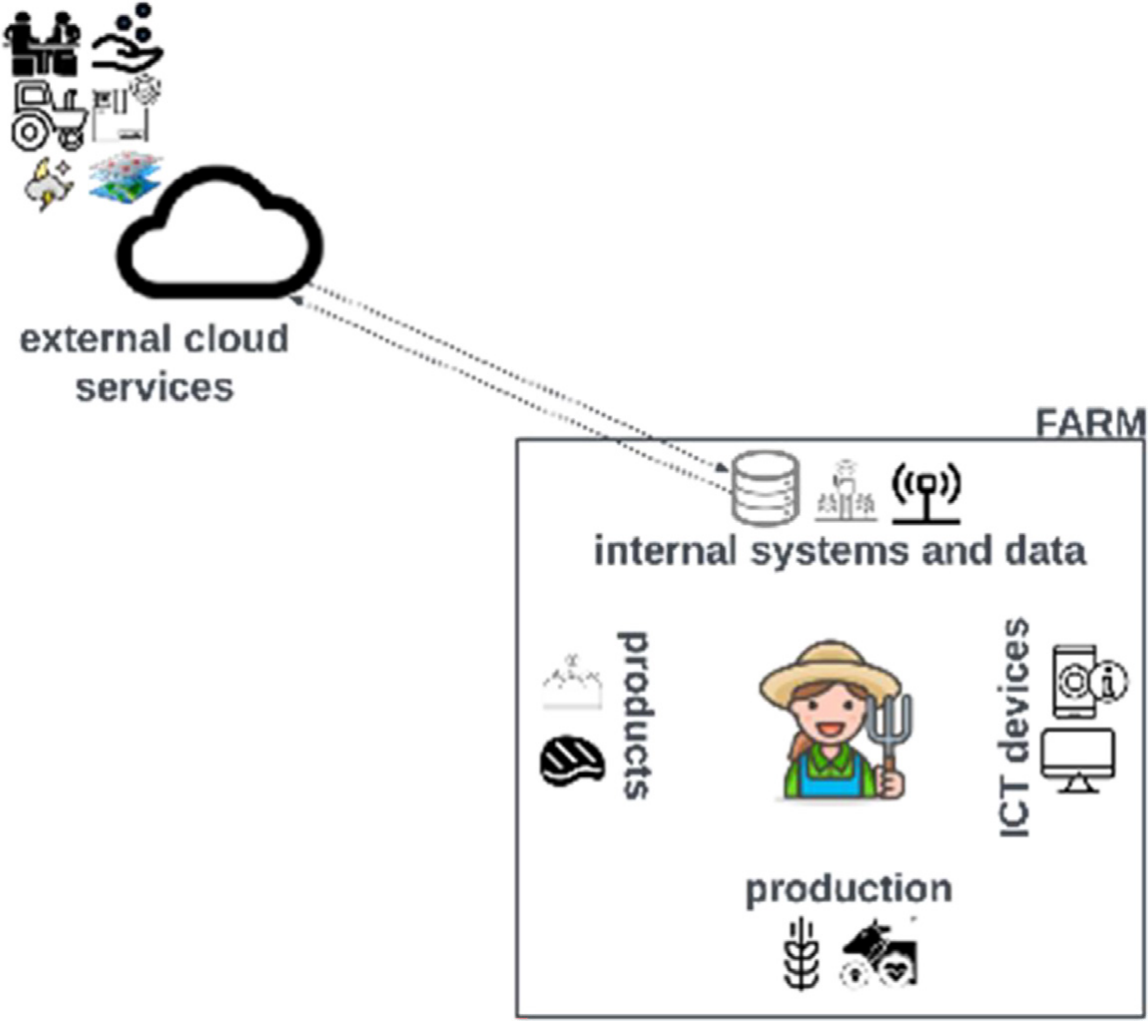
Farm data hosted on cloud services only.

We now examine the concept and potential of data spaces from the perspective of Antonio’s farm. Farm data is utilised by public administrations and actors in the supply chain, such as processors, certification bodies, and farmers’ associations. In Fig. 2, data are stored mainly on external cloud services run by private companies providing tools and services that Antonio bought to support digital data collection and management. However, these cloud-based systems often lack connections to national or regional authorities, leading to additional paperwork and costly duplication of work for farmers.

Currently, most farms rely on paper registries, and even digital applications in use cannot seamlessly exchange data with other applications. The subsequent step is the implementation of decision support systems to provide farmers with suggestions and insights through a software-as-a-service model. Many farms are already relying on digital systems and related data for their internal activities, with in situ or remote data collection stored in cloud services.

**Fig. 3:**
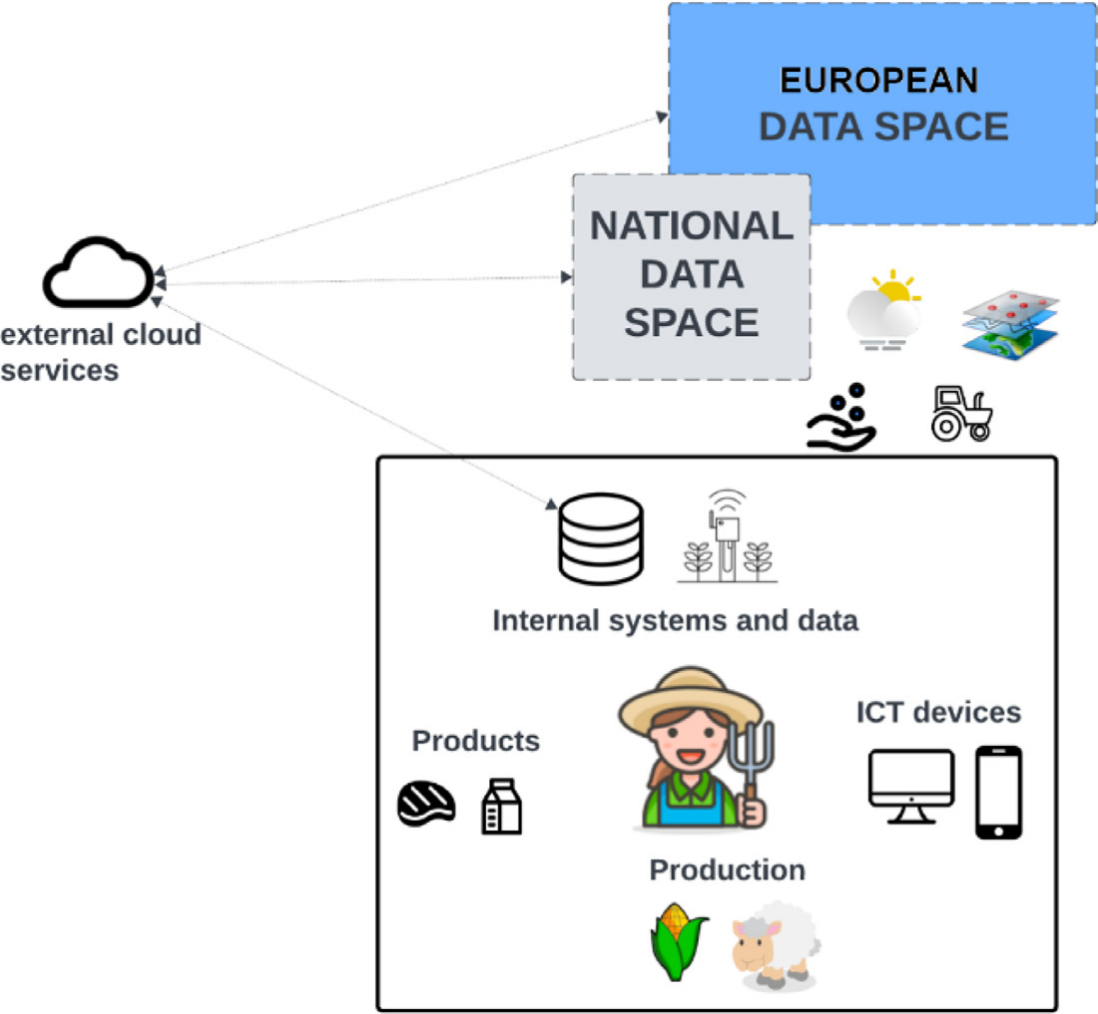
Farm data hosted also in national and Common European Data Spaces; services and applications use data from data spaces through cloud services.

In Fig. 3, we consider the introduction of data spaces at national and European levels, aiming to facilitate data exchange between national authorities and the EU for CAP-related data. This transition is expected to enhance interoperability for farmers by automating the feeding of national registries with required data, thus reducing duplication of work. Assuming that external cloud services are connected to the data spaces (using the aforementioned connectors) and share relevant data with e.g., control authorities, reporting activities can be partially automated. Farm data, already stored in cloud services, can be further shared with data spaces for different purposes; the assumption here is that specialised functionalities are made available by external service providers to connect directly to data spaces.

**Fig. 4:**
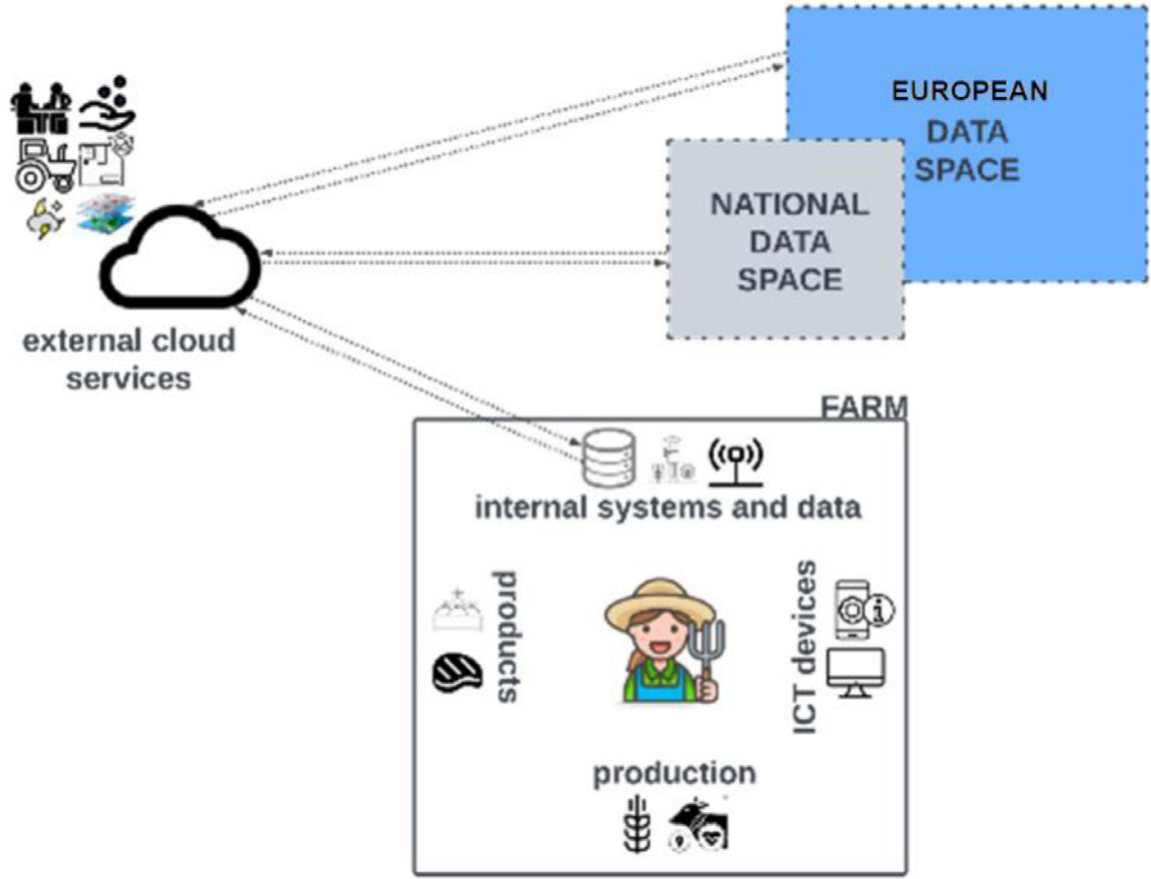
Potential future scenario envisaging services and applications directly connected to data spaces.

In Fig. 4, a potential future scenario is envisioned where most services and applications at the farm level directly connect to data spaces without the need for specialised functionalities made available by external service providers, thus reducing their dependence on other systems. This evolution allows for the creation of multiple user-driven spaces per domain, maximising potential for data producers and consumers. Data intermediaries are expected to play an increasingly crucial role in supporting users in accessing and using fragmented data dispersed across multiple sources, especially when it comes to data access control, consent giving, and data anonymisation activities. Data spaces could also support the implementation of the ‘once-only’ principle^14^ system, but its overall feasibility is debated [62].

We acknowledge here farmers’ concerns when it comes to automated data transfers because of a lack of digital skills to control the data exchanges, privacy concerns, fear of dependency on technology, costs, and complexity of both regulations and technology. The creation of data spaces could be a true opportunity to alleviate technology-related issues (e.g., lock-in, lack of interoperability, unclear data governance) but it must be reinforced by actions highlighting benefits and alleviating economic and regulatory-institutional barriers [63].

**Fig. 5:**
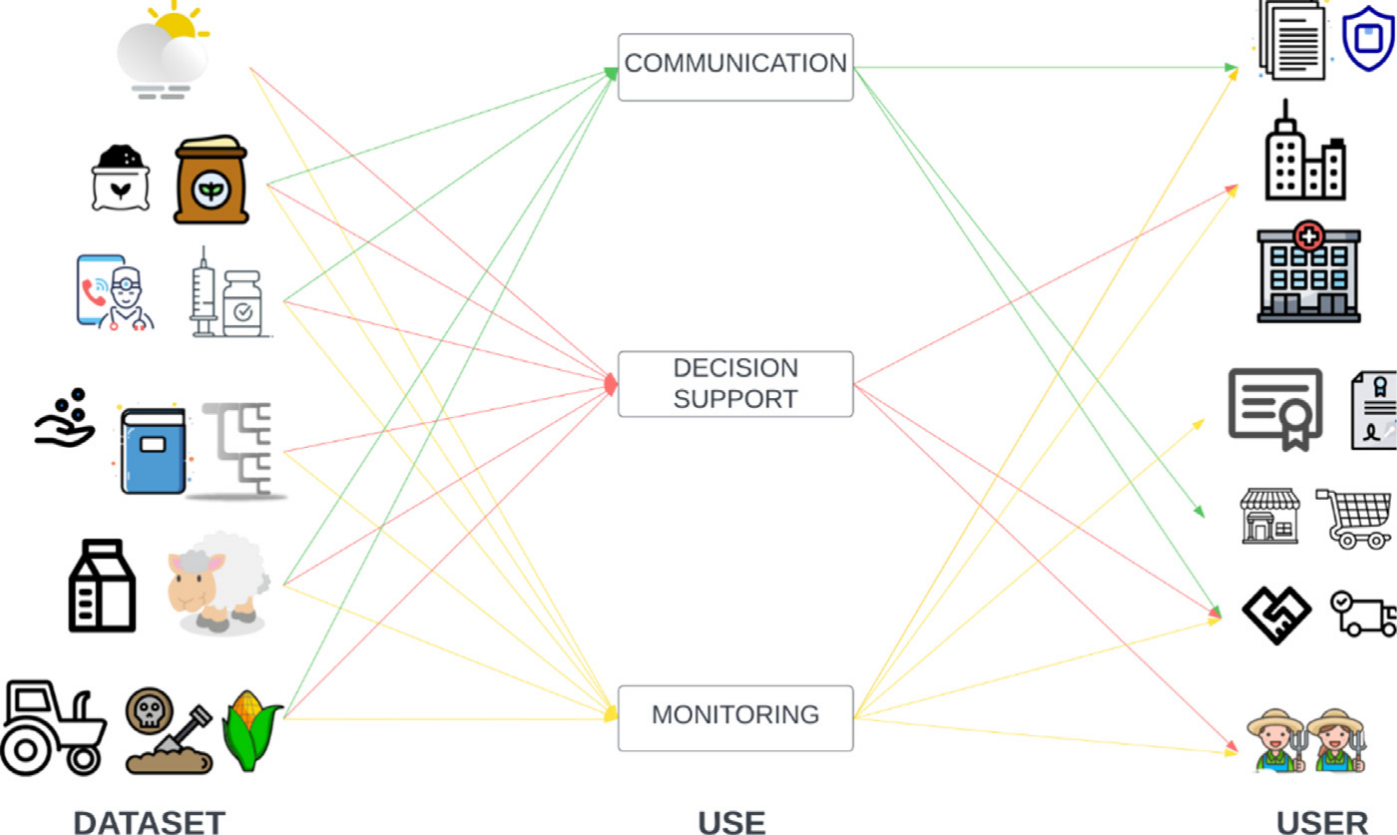
Links between datasets, use of data, and users.

Table 5 provides a series of examples of how data management can foster the creation of knowledge and related wisdom, which are also represented in Fig. 5 to highlight how different data sets contribute to different uses.

**Table 5:** Examples of knowledge created through data management.

ID	User	Knowledge	Wisdom
1,4,5	Farmer	Monitoring physiological and productive parameters of individuals and trend of production in time	Optimisation of the productivity
1,3	Farmer	Variation of herdcomposition in time. Seasonal patterns	Genetic selection
2,7,9	Farmer	Productivity of parcels	Optimisation of pasture sectionalisationand fertilisation
8	Cooperative	Analysis of farm’s needs	Technical support
1,5,6, 7,8,9	Consumer	Product traceability	Information to consumers
1,4	Cooperative	Assessment of potentialfarms annual production	Productionplanning
6	Health authority	Frequency of treatments, intensity of use of pharmaceuticals	Sanitary controls
7	Policy control boides	Frequency of treatments, intensity of use of fertilisers and pesticides	Compliance with agro-ecological schemes
1,5,7 8	Certification bodies	Origin of the milk, origin and quality of feed	Compliance with PDO
10	Consumer	Compliance with PDO	Information, informed choices
2,7	Regional authorities	Applied treatments	Pest alerts
2,4,5, 6,7,8 9	Insurance companies	Value of farm and products	Tailored prices of insurance products

In this use case, the journey towards an effective agri-food data space would involve enhancing interoperability, automating data sharing with public authorities, and reducing farmers’ administrative burden while increasing benefits for data producers and consumers. Although quite ambitious at the moment, such an objective should guide current developments in the sector.

## 6 Principles for an Agri-food Data Strategy

The establishment of an agri-food data space is a complex undertaking, as it must address numerous economic, legal, technical, motivational, and governance barriers. To motivate all stakeholders, strategies for data spaces should be accompanied by compelling evidence of benefits that exceed associated costs. When interpreting data spaces as tools to access existing data from various sources to generate insights for different purposes and user groups, several critical questions arise.

Ensuring fair data sharing and ethical utilisation of data, as well as preventing data analysis skills and access to big data from favouring selected players, are key concerns. Anonymising data sufficiently to avoid revealing business secrets and motivating data sharing from smart devices used across the food chain are also crucial. Moreover, ensuring that data can be transformed into information for different purposes with specific degrees of aggregation, data quality, and resolution that suit relevant use cases is essential. Additionally, enabling farmers to interpret and utilise large-scale data for small-scale applications, and facilitating access to useful decision support for strategic decisions, are significant challenges.

Initiatives to integrate data collection for administrative controls and business could be coordinated but kept separate, with commonly agreed rules stating which of the voluntarily shared data might be used for other purposes (such as research, commerce by third parties or administrative controls). To build trust, farmers could be encouraged to share their data rather than forced to. On the other hand, governance frames might address concerns related to open data access, competition, surveillance, and stigma [64]. The role of Agricultural Knowledge and Innovation Systems (AKIS) is pivotal in farm data collection and storage, and the digitalization of extension services could transform AKIS into farmer-centred digital ecosystems [65], [66], [67].

Furthermore, the process of constructing data spaces might involve a mix of bottom-up and top-down initiatives, linking the co-development of digital ecosystems with the construction of data spaces. Bottom-up initiatives should focus on real-life experiments and the development of use cases, generating a critical mass of actors, tools, and institutions to test data spaces in various contexts. Strengthening weaker sectors and areas and addressing the digital divide should be a specific focus, accompanied by innovation policies and research supporting these initiatives [68], [69]. Governance issues, quality assurance, and ethics of digital ecosystems could also be addressed, with specific attention given to the available skills at the local level. Complementing bottom-up initiatives, top-down frameworks could provide rules, standards, and tools to favour interoperability and adaptability to different contexts [70]. Regulation could contribute to specific issues such as ownership and access, which in the Data Act are still left to private contracts [18]. We acknowledge here the debate in the literature regarding the Data Act, a cross-sectoral piece of legislation laying out principles and guidelines for all sectors, that considers how remaining issues may need to be addressed by follow-up sectoral regulation [71].

Overall, the construction of data spaces requires a comprehensive approach that addresses a multitude of technical, governance, and ethical challenges while delivering clear benefits to farmers and stakeholders. This approach should involve a careful balance of bottom-up experimentation and top-down frameworks to ensure that data spaces are efficient, accessible, and (co-)beneficial for all stakeholders.

## 7 Concluding Remarks

Drawing on a conceptual framework that considers data as a component of a ’knowledge hierarchy’, this paper has discussed the functions that data can perform for different types of actors. To be turned into knowledge and wisdom, data need users able to use them in specific contexts.

The European Data Spaces initiative is a strong opportunity to encourage the blossoming of context-sensitive data spaces centered on the needs of farmers, especially small and medium-sized ones. The role of data held by public authorities, in this regard, can be an important driver, as the integration of public data with farm and supply chain data can show farmers the value of data, and generate a wide range of services in the context of AKIS.

Given the specificities of agri-food European contexts, we have stressed that a European Data Space should be based on a network of participatory context-sensitive data spaces. Each of them could operate with rules that allow the interoperability of data and compliance with public purposes, but their regulation and governance should be based on the specific needs of the users in each context. A bottom-up process of construction would allow experimentation based on local needs, and (co-) learning processes could demonstrate the added value of data through use cases, providing insight for further exploration by all stakeholders. The framework we propose links the building of data spaces to the support of the development of digital ecosystems where the production, the use, and the exchange of data occur. This would help address a chicken-egg problem, as without data, digital services cannot take off, and without digital services, there is no motivation to share the data.

We would like to recall the lessons learned in the INSPIRE implementation again, highlighting how the process should be demand-driven, fuelled by the evidence of clear benefits, and evidence-based for stakeholders relying on data and data spaces. Thus, continuing the stocktaking activity initiated with Horizon Europe to gain better insights into the data value in the agri-food system is of utmost importance. Moreover, a sector-specific regulatory initiative, guiding and supporting standard development and governance, will help overcome the complexity of the sector -facing challenges such as strong fragmentation-, diversity and disparity of the actors, the multiplicity of policy areas, and different interests linked to data access and use.

## CRediT authorship contribution statement

**Gianluca Brunori:** Conceptualization, Data curation, Investigation, Methodology, Writing – original draft, Writing – review & editing. **Manlio Bacco:** Conceptualization, Data curation, Investigation, Methodology, Visualization, Writing – original draft, Writing – review & editing. **Carolina Puerta-Piñero:** Conceptualization, Investigation, Visualization, Writing – review & editing. **Maria Teresa Borzacchiello:** Conceptualization, Data curation, Investigation, Validation, Writing – review & editing. **Eckhard Stormer:** Conceptualization, Investigation, Validation, Writing – review & editing.

## Conflict of Interest

None.

## Footnotes

2 ‘Data holder’ means a legal person, including public sector bodies and international organisations, or a natural person who is not a data subject with respect to the specific data in question, which, in accordance with applicable Union or national law, has the right to grant access to or to share certain personal or non-personal data [12].

3 Regulation on data governance – Questions and Answers - Brussels, 25 November 2020 https://ec.europa.eu/commission/presscorner/detail/en/QANDA_20_2103.

4 DIH AGRIFOOD Dataspace, https://itc-cluster.com/dih-agrifood-dataspace.

5 https://agdatahub.eu.

6 https://www.djustconnect.be.

7 https://www.data-avaruus.fi/fi/afdsf-uutiskirjeet.

8 https://www.agronod.com.

9 https://www.sitra.fi/en/projects/pilot-projects-gaia-x-finland/#results-in-brief.

10 GA 818194, https://desira2020.agr.unipi.it.

11 GA 101083401, https://agridataspace-csa.eu.

12 https://open-science-cloud.ec.europa.eu.

13 We point out how the aforementioned EU Code of Conduct for Agricultural Data Sharing explicitly states that the data owner is to be considered the data generator (“the rights regarding data produced on the farm or during farming operations are granted to (’owned by’) the farmer and may be used extensively by them.”). The Data Act instead considers as the data holder the subject (e.g., the manufacturer of smart machinery) with the power to grant access to data to users (e.g., the farmer), with the possibility (not the right) to manage permissions related to access to their data by third parties.

14 https://ec.europa.eu/digital-building-blocks/sites/display/DIGITAL/Once+Only+Technical+System.

## References

[1] FAO (Food and Agriculture Organization of the United Nations), Developing sustainable food value chains. guiding principles, 2014.

[2] FAO(Food and Agriculture Organization of the United Nations), Sustainable food systems: concept and framework, 2018.

[3] Commission E., Research D.-G.f., Innovation, Advisors G.o.C.S. (2023).

[4] COMMISSION E. (2020). Farm to fork strategy: for a fair, healthy and environmentally-friendly food system. Communication from the Commission to the European Parliament, the Council, the European Economic and Social Committee and the Committee of the Regions.

[5] Schebesta H., Candel J.J.L. (2020). Game-changing potential of the EU’s farm to fork strategy. Nat. Food.

[6] EUROSTAT, Farms and farmland in the european union—statistics (2022).

[7] World Bank, World development report 2021: Data for better lives, 2021.

[8] Fanzo J., Haddad L., Schneider K.R., Béné C., Covic N.M., Guarin A., Herforth A.W., Herrero M., Sumaila U.R., Aburto N.J. (2021). Rigorous monitoring is necessary to guide food system transformation in the countdown to the 2030 global goals. Food Policy.

[9] European Parliament, The Council, Regulation (EU) 2016/679 of the european parliament and of the council of 27 April 2016 on the protection of natural persons with regard to the processing of personal data and on the free movement of such data, and repealing directive 95/46/EC (general data protection regulation), OJ 2016 l 119/1 (2016).

[10] European Parliament, The Council, Directive of the european parliament and of the council on open data and the re-use of public sector information (recast)(2019).

[11] COMMUNICATIONFROMTHECOMMISSIONTOTHEEUROPEANPARLIAMENT, THE COUNCIL, THE EUROPEAN ECONOMIC AND SOCIAL COMMITTEE AND THE COMMITTEE OF THE REGIONS, T. COUNCIL, T.E.E. REGIONS, S. COMMITTEE, T.C.O.F. THE, A European strategy for data, 2020.

[12] REGULATION(EU) 2022/868 OF THE EUROPEAN PARLIAMENT AND OF THE COUNCIL, O.F. THE, European data governance and amending Regulation (EU) 2018/1724 (Data Governance Act), 2022.

[13] REGULATION(EU) 2023/2854 OF THE EUROPEAN PARLIAMENT AND OF THE COUNCIL, harmonised rules on fair access to and use of data and amending Regulation (EU) 2017/2394 and Directive (EU) 2020/1828 (Data Act), 2023.

[14] Farrell E., Minghini M., Kotsev A., Soler Garrido J., Tapsall B., Micheli M., Posada Sanchez M., Signorelli S., Tartaro A., Bernal Cereceda J. (2023). Technical Report.

[15] Pol E. (2023). Technical Report.

[16] (2024). Technical Report.

[17] Kotsev A., Minghini M., Tomas R., Cetl V., Lutz M. (2020). From spatial data infrastructures to data spaces—A technological perspective on the evolution of european SDIs. ISPRS Int. J. Geo-Inf..

[18] Atik C. (2022). Towards comprehensive European agricultural data governance: moving beyond the “data ownership” debate. IIC-Int. Rev. Intellectual Property Competition Law.

[19] Curry E., Scerri S., Tuikka T. (2022).

[20] Wilkinson M.D., Dumontier M., Aalbersberg I.J., Appleton G., Axton M., Baak A., Blomberg N., Boiten J.-W., da Silva Santos L.B., Bourne P.E. (2016). The FAIR guiding principles for scientific data management and stewardship. Sci. Data.

[21] Regulation (EU) 2024/903 of the european parliament and of the council of 13 March 2024 laying down measures for a high level of public sector interoperability across the union (interoperable europe act), 2024.

[22] Ackoff R.L. (1989). From data to wisdom. J. Appl. Syst. Anal..

[23] V. Cetl, M. Nunes de Lima, R. Tomas, M. Lutz, J. D’Eugenio, A. Nagy, J. Robbrecht, Summary report on status of implementation of the INSPIRE directive in EU (2017).

[24] Ponti M., Portela M., Pierri P., Daly A., Milan S., Kaukonen Lindholm R., Maccani G., Peter De Souza S., Thabit Gonzalez S. (2024). Technical Report.

[25] Newell P. (2008). Civil society, corporate accountability and the politics of climate change. Glob. Environ. Polit..

[26] Jandrić P. (2020). Postdigital research in the time of covid-19. Postdigital Sci. Educ..

[27] Coble K.H., Mishra A.K., Ferrell S., Griffin T. (2018). Big data in agriculture: a challenge for the future. Appl. Econ. Perspect. Policy.

[28] M. Micheli, E. Farrell, S.B. Carballa, S.M. Posada, S. Signorelli, M. Vespe, et al., Mapping the landscape of data intermediaries, 2023.

[29] Rolandi S., Brunori G., Bacco M., Scotti I. (2021). The digitalization of agriculture and rural areas: towards a taxonomy of the impacts. Sustainability.

[30] Remondino M., Zanin A. (2022). Logistics and agri-food: digitization to increase competitive advantage and sustainability. literature review and the case of italy. Sustainability.

[31] Kenney M., Serhan H., Trystram G. (2020). Digitization and platforms in agriculture: organizations, power asymmetry, and collective action solutions. Power Asymmetry, and Collective Action Solutions (June 20, 2020).

[32] IoT Business News, The precision agriculture market to reach € 5.2 billion worldwide in 2027, 2023. https://iotbusinessnews.com/2023/12/22/53545-the-precision-agriculture-market-to-reach-e-5-2-billion-worldwide-in-2027.

[33] Statista, Forecast market value of smart farming worldwide in 2021 to 2027, 2023. https://www.statista.com/statistics/720062/market-value-smart-agriculture-worldwide.

[34] Miranda J., Ponce P., Molina A., Wright P. (2019). Sensing, smart and sustainable technologies for agri-food 4.0. Comput. Ind..

[35] Kalmar R., Rauch B., Dörr J., Liggesmeyer P. (2022).

[36] C. Atik, Data access problems in the emerging digital agriculture sector: what role for EU competition law enforcement and regulatory intervention? (2023).

[37] Stachowicz J., Umstätter C. (2021). Do we automatically detect health-or general welfare-related issues? A framework. Proc. R. Soc. B.

[38] Purcell W., Neubauer T. (2023). Digital twins in agriculture: a state-of-the-art review. Smart Agric. Technol..

[39] Verdouw C.N., Wolfert J., Beulens A., Rialland A. (2016). Virtualization of food supply chains with the internet of things. J. Food Eng..

[40] Oncini F., Bozzini E., Forno F., Magnani N. (2020). Towards food platforms? An analysis of online food provisioning services in Italy. Geoforum.

[41] Parker G.G. (2016).

[42] Bustamante M.J. (2023). Digital platforms as common goods or economic goods? Constructing the worth of a nascent agricultural data platform. Technol. Forecast. Soc.Change.

[43] Goasduff L. (2021).

[44] Metta M., Ciliberti S., Obi C., Bartolini F., Klerkx L., Brunori G. (2022). An integrated socio-cyber-physical system framework to assess responsible digitalisation in agriculture: a first application with living labs in europe. Agric. Syst..

[45] E.V. de Velde, D. Kretz, Advanced technologies for industry – sectoral watch (technological trends in the agri-food industry), 2020.

[46] O’Kane G. (2012). What is the real cost of our food? Implications for the environment, society and public health nutrition. Public Health Nutr..

[47] Hassoun A., Boukid F., Pasqualone A., Bryant C.J., García G.G., Parra-López C., Jagtap S., Trollman H., Cropotova J., Barba F.J. (2022). Emerging trends in the agri-food sector: Digitalisation and shift to plant-based diets. Curr. Res. Food Sci..

[48] Ehlers M.-H., Huber R., Finger R. (2021). Agricultural policy in the era of digitalisation. Food Policy.

[49] Finck M., Mueller M.-S. (2023). Access to data for environmental purposes: setting the scene and evaluating recent changes in EU data law. J. Environ. Law.

[50] Kotsev A., Minghini M., Cetl V., Penninga F., Robbrecht J., Lutz M. (2021). Inspire—A public sector contribution to the european green deal data space. A vision for the technological evolution of Europe’s Spatial Data Infrastructures for.

[51] Cammarano D., Olesen J.E., Helming K., Foyer C.H., Schönhart M., Brunori G., Bandru K.K., Bindi M., Padovan G., Thorsen B.J. (2023). Models can enhance science–policy–society alignments for climate change mitigation. Nat. Food.

[52] Arts K., Van der Wal R., Adams W.M. (2015). Digital technology and the conservation of nature. Ambio.

[53] Van Der Velden D., Dessein J., Klerkx L., Debruyne L. (2023). Constructing legitimacy for technologies developed in response to environmental regulation: the case of ammonia emission-reducing technology for the flemish intensive livestock industry. Agric. Human Values.

[54] Šestak M., Copot D. (2023). Towards trusted data sharing and exchange in agro-food supply chains: design principles for agricultural data spaces. Sustainability.

[55] Carletto C. (2021). Better data, higher impact: improving agricultural data systems for societal change. Eur. Rev. Agric. Econ..

[56] Vicente-Saez R., Gustafsson R., Van den Brande L. (2020). The dawn of an open exploration era: emergent principles and practices of open science and innovation of university research teams in a digital world. Technol. Forecast. Soc. Change.

[57] Evans J.A., Foster J.G. (2011). Metaknowledge. Science.

[58] Vicente-Saez R., Martinez-Fuentes C. (2018). Open science now: a systematic literature review for an integrated definition. J. Bus. Res..

[59] Bonney R., Shirk J.L., Phillips T.B., Wiggins A., Ballard H.L., Miller-Rushing A.J., Parrish J.K. (2014). Next steps for citizen science. Science.

[60] Burgelman J.-C., Pascu C., Szkuta K., Von Schomberg R., Karalopoulos A., Repanas K., Schouppe M. (2019). Open science, open data, and open scholarship: European policies to make science fit for the twenty-first century. Front. Big Data.

[61] Wiseman L., Sanderson J., Zhang A., Jakku E. (2019). Farmers and their data: an examination of farmers’ reluctance to share their data through the lens of the laws impacting smart farming. NJAS-Wageningen J. Life Sci..

[62] Stiefel L. (2022). An issue of data: a digital platform unsuitable for swiss agriculture. Etudes Rurales.

[63] Ferrari A., Bacco M., Gaber K., Jedlitschka A., Hess S., Kaipainen J., Koltsida P., Toli E., Brunori G. (2022). Drivers, barriers and impacts of digitalisation in rural areas from the viewpoint of experts. Inf. Softw. Technol..

[64] O.E.C.D., Digital Opportunities for Better Agricultural Policies, 2019.

[65] Fielke S., Taylor B., Jakku E. (2020). Digitalisation of agricultural knowledge and advice networks: a state-of-the-art review. Agric. Syst..

[66] Ingram J., Maye D. (2020). What are the implications of digitalisation for agricultural knowledge?. Front. Sustain. Food Syst..

[67] Kiraly G., Vago S., Bull E., van der CRUYSSEN L., Arbour T., Spanoghe P., van Dijk L. (2023). Information behaviour of farmers, foresters, and advisors in the context of digitalisation in the EU. Stud. Agric. Econ..

[68] Cronin E., Fosselle S., Rogge E., Home R. (2021). An analytical framework to study multi-actor partnerships engaged in interactive innovation processes in the agriculture, forestry, and rural development sector. Sustainability.

[69] Duncan J., DeClerck F., Báldi A., Treyer S., Aschemann-Witzel J., Cuhls K., Ahrné L., Bisoffi S., Grando S., Guobys L. (2022). Democratic directionality for transformative food systems research. Nat. Food.

[70] Brown H.S., Vergragt P.J. (2008). Bounded socio-technical experiments as agents of systemic change: the case of a zero-energy residential building. Technol. Forecast. Soc. Change.

[71] Can A. (2023). Horizontal intervention, sectoral challenges: evaluating the data act’s impact on agricultural data access puzzle in the emerging digital agriculture sector. Comput. Law Secur. Rev..

